# A Novel Covalent Inhibitor Fragment for the SARS-CoV‑2 Main Protease Identified by Target-Specific Deep Learning

**DOI:** 10.1021/acschembio.6c00120

**Published:** 2026-05-01

**Authors:** Weijun Zhou, Angel D′Oliviera, Xuhang Dai, Jeffrey S. Mugridge, Yingkai Zhang

**Affiliations:** † Department of Chemistry, 5894 New York University, New York, New York 10003, United States; ‡ Department of Chemistry & Biochemistry, 5972 University of Delaware, Newark, Delaware 19716, United States; § Simons Center for Computational Physical Chemistry at New York University,New York, New York 10003, United States; ∥ NYU-ECNU Center for Computational Chemistry at NYU Shanghai, Shanghai 200062, China

## Abstract

The SARS-CoV-2 main protease (M^pro^, also known as 3CL^pro^) is an attractive antiviral drug target due to its essential role in viral replication and absence of human homologues. Development of new coronavirus-specific M^pro^ inhibitors will be important as SARS-CoV-2 continues to evolve. Leveraging the rapidly expanding pool of diverse, experimental M^pro^-inhibitor data, we developed a target-specific deep learning workflow to accelerate the discovery of new M^pro^ inhibitor compounds and fragment-like starting points. This workflow combined a fine-tuned inhibitor prediction model with solubility (logS) and lipophilicity (logP) models, molecular similarity analysis, and literature mining to prioritize novel, drug-like candidates. Applied to a purchasable library of over 500,000 compounds, the approach rapidly identified 24 candidates for experimental testing. Biochemical assays revealed a novel, small covalent inhibitor fragment (A02) with an apparent IC_50_ of 1.5 μM, prior to any synthetic optimization or derivatization. A 1.76 Å crystal structure of M^pro^ bound to A02 confirmed covalent modification of the catalytic M^pro^ cysteine (C145), unique engagement of the underutilized M^pro^ S3′ pocket, and the potential for derivatives of this scaffold to interact with additional M^pro^ pockets in future optimization efforts. Together, these results demonstrate the potential for target-specific deep learning approaches to guide the rapid screening and discovery of new inhibitor leads or drug scaffolds.

## Graphical abstract

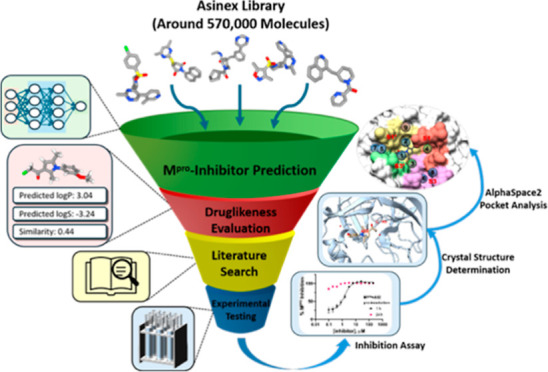

## 1 Introduction

Target-specific deep learning approaches have emerged as powerful tools in accelerating drug discovery by tailoring predictive computational models to individual biological targets.^1^ Targeted approaches to inhibitor design leverage curated data sets including biochemical assay results, crystallographic structures, and inhibitor profiles to capture fine-grained structure–activity relationships (SARs) that improve model selectivity and predictive accuracy, and thereby enable more efficient virtual screening, de novo molecular design, and lead optimization. For example, Zhavoronkov et al.^2^ recently used a reinforcement learning model pretrained on ∼17,000 kinase-inhibitor records and fine-tuned on target-specific data to generate 30,000 predicted DDR1 kinase inhibitor structures, from which six compounds were synthesized, four identified to have biochemical activity, two validated in cells, and one found to have favorable mouse pharmacokinetics. By using these target-specific learning models, kinase inhibitors were able to be rapidly screened, identified, and validated in only two months’ time. Similar computational approaches have produced libraries of target specific scaffolds for diverse enzymes and receptors,^3^ submicromolar inhibitors for kinases, and promising drug repositioning candidates for Alzheimer’s disease.^4^ Together, these examples illustrate how optimizing deep-learning workflows on target-specific data delivers higher selectivity, and faster iteration cycles than generalized models in order to rapidly identify high-quality candidate inhibitor molecules.

Despite the huge success of COVID-19 vaccines, SARS-CoV-2 and related coronaviruses continue to pose a serious threat to immunocompromised individuals and remain a potential source of future pandemics.^5,6^ The coronavirus main protease (M^pro^, also known as 3CL^pro^) is essential for processing the viral polyproteins required for replication, is highly conserved among coronaviruses, and has no close human homologues, making it an attractive antiviral target with low off-target risks.^7^ Nirmatrelvir is a potent inhibitor of the SARS-CoV-2 main protease that has been clinically effective and widely used in treating SARS-CoV-2 infection, but many Nirmatrelvir resistance mutations have already been detected in patients and are likely to continue evolving,^8–11^ underscoring the need for novel coronavirus inhibitor scaffolds. In this study, we leverage the extensive biochemical data now available for SARS-CoV-2 M^pro^ inhibition to enable a target-specific deep learning approach aimed at identifying new M^pro^ inhibitor scaffolds or chemotypes.

The recent availability of extensive SARS-CoV-2 M^pro^ experimental data sets, including high-throughput screening results and fragment-based crystallography, has opened the door for developing target-specific deep learning models of M^pro^ inhibition. For example, Jin et al.^7^ screened over 10,000 compounds against M^pro^, identifying potent inhibitors through structure-based virtual screening and fluorescence resonance energy transfer (FRET) assays. Similarly, Douangamath et al.^12^ employed fragment-based screening to uncover high-value fragment hits binding to the M^pro^ active site. These studies contributed to the rapidly expanding pool of experimental data that sets the stage for target-specific machine learning model development for rapid M^pro^ inhibitor discovery. Along these lines, several recent reports have used computational approaches including a gradient-boosted decision trees (GBDT) model^13^ and integrated deep reinforcement learning^14^ to predict existing drug molecules that might be potent inhibitors of M^pro^ and to generate noncovalent M^pro^ binders with low micromolar IC_50_ values.

Here, we report the discovery of a novel covalent M^pro^ inhibitor scaffold identified using a target-specific deep learning workflow trained on temporally split M^pro^-inhibitor data sets. This approach better mimics the real-world drug discovery process by incorporating data from across the pandemic timeline, which enabled the rapid prediction and prioritization of candidate compounds. Our temporal deep learning strategy generated 24 high-priority candidate M^pro^ inhibitors and after biochemical screening we identified and validated a small molecule covalent inhibitor fragment (A02) with an apparent IC_50_ value of 1.5 μM prior to any optimization. A 1.76 Å crystal structure of M^pro^-A02 revealed key covalent and noncovalent interactions between A02 and M^pro^, including A02’s engagement with the underutilized M^pro^ S3′ pocket. Further structural analysis of A02 binding and M^pro^ pocket occupancy suggests that A02 has potential for further optimization as a novel M^pro^ inhibitor scaffold.

## 2 Results and Discussion

In this study, we developed and optimized a series of KANO^15^ (knowledge graph-enhanced molecular contrastive learning with functional prompt) models to accurately predict key molecular properties, which enabled the virtual screening of the Asinex Virtual Library^16^ for potential SARS-CoV-2 M^pro^ inhibitors. Our fine-tuned models demonstrated high precision and robust performance in predicting solubility and lipophilicity, establishing a reliable framework for compound evaluation. The subsequent application of these models led to the identification of 24 potential M^pro^ inhibitors. Experimental inhibition assays confirmed the potent, time-dependent inhibitory activity of compound A02, which was further validated as a covalent inhibitor through detailed structural analysis revealing its direct modification of the M^pro^ active site catalytic cysteine residue. Additionally, our biochemical and structural assessment highlights A02 as a unique M^pro^ inhibitor scaffold compared to previously reported compounds, and the M^pro^-A02 AlphaSpace2 pocket analysis^17^ revealed significant opportunities for further optimization of A02 interactions with multiple adjacent M^pro^ substrate binding pockets to develop high-affinity and high-selectivity antiviral drugs based on the A02 chemotype.

### 2.1 Development and Assessment of KANO Fine-Tuned Models

To proceed with virtual screening and identify potential M^pro^ inhibitor candidates, we developed three KANO-based models for virtual screening: an M^pro^-inhibitor classification model, a logS regression model, and a log *P* regression model. The M^pro^-inhibitor model was trained on 13,484 curated compounds from the NIH 3CL assay,^18^ COVID Moonshot,^19^ and literature sources, while the logS and logP models were developed using the AQUA/ESOL/PHYS^20–22^ and PHYSPROP^23^ data sets, respectively. Details of data set curation, split strategy, and model evaluation are provided in Section S5 and the Supporting Information. These properties—biological activity, solubility, and lipophilicity—are critical for selecting compounds with both therapeutic potential and suitable drug-like characteristics. The main classification model, KANO-M^pro^-inhibitor, was developed to identify likely M^pro^ inhibitors and achieved a precision of 0.883, meaning that nearly 88% of the compounds it predicted as active were indeed true inhibitors in retrospective tests. Although its recall was lower at 0.308 (Table S3), we found that attempts to improve recall through resampling yielded only modest gains while substantially reducing precision, thereby increasing the number of false positives advanced for experimental testing (Figure 1A). In virtual screening, high precision is particularly important because predicted hits often need to be purchased or synthesized for follow-up experimental testing—a process that can be costly and time-consuming. By minimizing false positives, we reduce wasted resources and experimental effort. Definitions of performance metrics and training details are described in the Materials and Methods. In addition, two regression models were developed to predict solubility (KANO-logS) and lipophilicity (KANO-log P), which are standard indicators of a compound’s suitability for oral bioavailability and formulation. The KANO-logS model achieved a root-mean-square error (RMSE) of 0.78 and a Pearson correlation coefficient (PCC) of 0.94 on a large data set, showing strong agreement between predicted and experimental solubility values (Figure 1C,D). Similarly, the KANO-logP model achieved an RMSE of 0.366, outperforming several previously reported computational approaches for logP prediction (Table S4), including sPhysNet-MT-ens5, QSPR, GraphCNN, DNNtaut, DNNmono, and OPERA,^24^ indicating accurate prediction of lipophilicity (Figure 1B). These two models support downstream filtering of candidate compounds based on drug-likeness criteria typically used in early phase screening.

**Figure 1:**
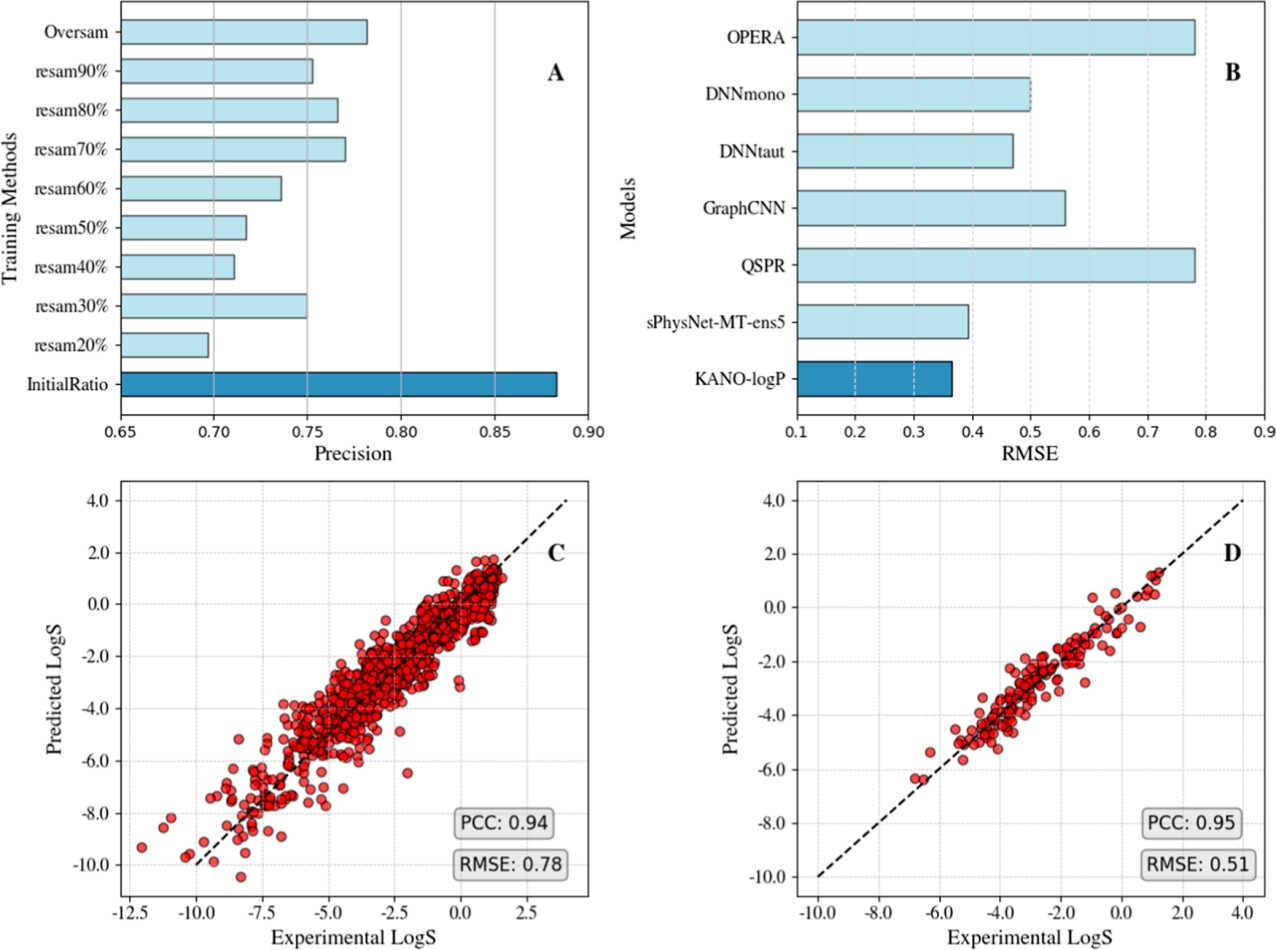
Performance of fine-tuned KANO Models. (A) KANO-M^pro^-inhibitor model performance on the test set. (B) KANO-logP Model: RMSE (*x*-axis) vs models (*y*-axis). (C) KANO-logS Model (1204 Compounds derived from the unique records in the AQUA, ESOL, and PHYS data sets): experimental logS (*x*-axis) vs predicted logS (*y*-axis). (D) KANO-logS Model (subset of 62 approved drugs with experimental logS from DrugBank): experimental logS (*x*-axis) vs predicted logS (*y*-axis).

To further improve the inhibitor model’s performance despite class imbalance (few actives vs many inactives), we tested different training strategies. While reducing inactive samples improved sensitivity to actives, it also increased the number of false positives. Retaining the original data distribution ultimately gave the best precision, which is critical for guiding experimental validation. Taken together, these models provide a data-driven framework to prioritize compounds that not only show predicted inhibitory activity but also possess favorable solubility and lipophilicity profiles—helping to bridge the gap between computational predictions and practical experimental testing (Table S3).

### 2.2 Virtual Screening Workflow Results

The selected models were applied to the Asinex Virtual Library^16^ (575,302 compounds), predicting 425 compounds as active M^pro^-inhibitors. These compounds were then subjected to downstream filtering based on drug-likeness and novelty criteria, including predicted logS (−5 to 0), predicted logP (0 to 5), and Tanimoto similarity (Tc) < 0.85. For the novelty filter, Tc was calculated using RDKit Morgan fingerprints by comparing each predicted positive compound against our curated Mpro-inhibitor data set and PubChem M^pro^-related compounds (19,677 records). A subsequent literature search was used to further assess novelty, leaving 76 compounds after exclusion of previously reported or tested compounds. These remaining compounds were then ranked by predicted probability of M^pro^ inhibition, and the top 24 candidates were selected for bioassay testing. The average probability of these candidates being active M^pro^-inhibitors was 0.625, with average predicted logP and logS values of 3.13 and −4.39, respectively. The average Tc of 0.388 also indicated low similarity between these candidates and existing M^pro^-inhibitor data. The predicted properties of the 24 selected drug candidates are presented in Table S5, and the workflow is summarized in Figure 2.

**Figure 2:**
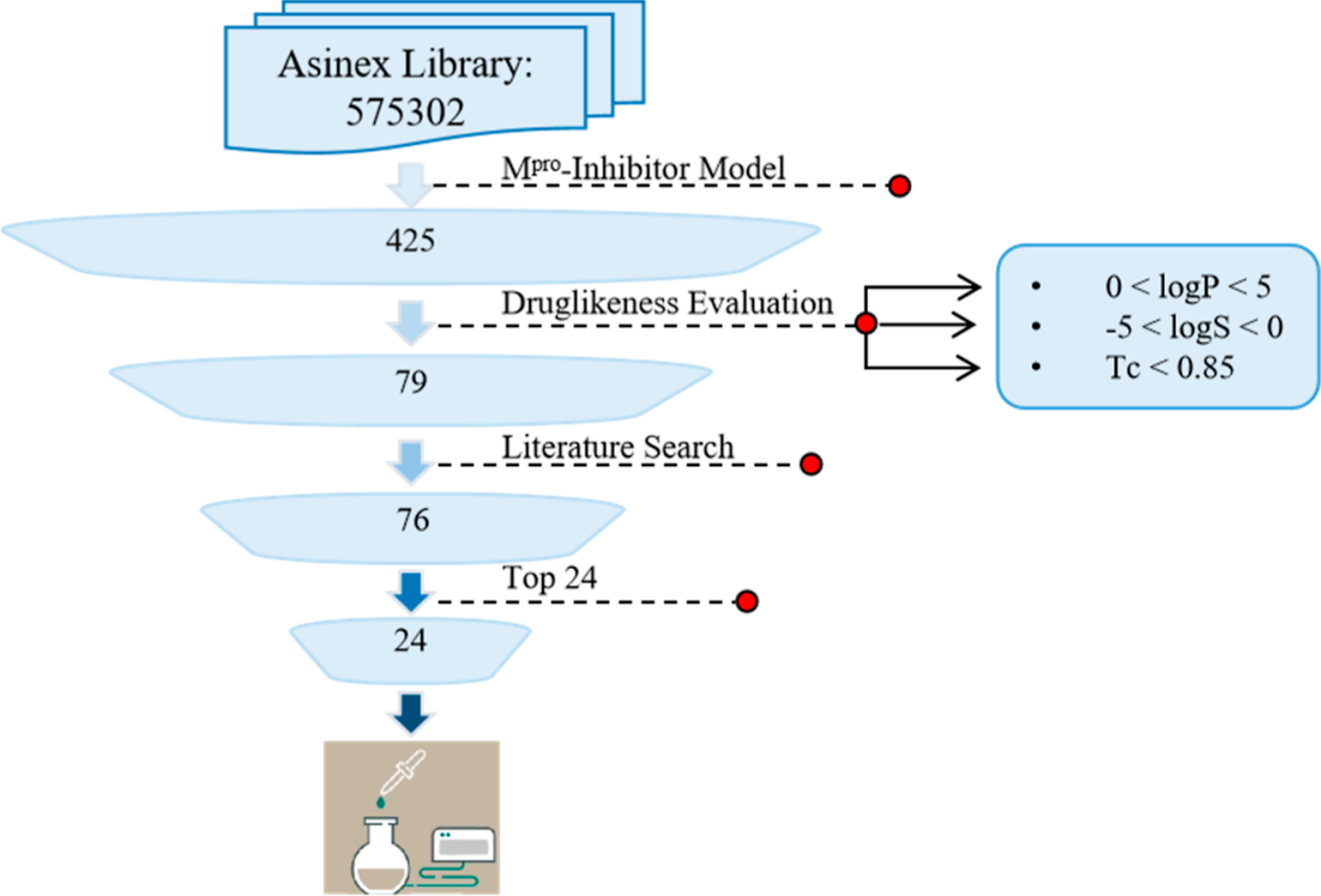
Workflow Summary. Step 1: M^pro^-inhibitor model—Compounds with a predicted probability of less than 0.5 are filtered out. Step 2: drug-likeness Evaluation—compounds that do not meet the logS, logP, or novelty criteria are removed. Step 3: literature search—compounds that have already been tested on M^pro^ inhibitors (including SARS-CoV-2 and SARS-CoV-1) are excluded. Three compounds were excluded because they were evaluated against SARS-CoV-1 and exhibited inactive activity. Step 4: final Top 24 compounds—the top 24 compounds with the highest predicted probability of being active M^pro^-inhibitors are selected for further testing.

### 2.3 Experimental M^pro^ Inhibition Screen

To evaluate the M^pro^ inhibition properties of the top 24 drug candidates (A01–A24, Figure S8) selected from the above virtual screening method, we expressed and purified SARS-CoV-2 M^pro^ and monitored the cleavage of a prototypical, quenched fluorogenic peptide substrate in the presence of either 20 or 200 μM of the candidate inhibitors (Figure 3A). For these initial inhibition screens, M^pro^ and the candidate inhibitor were incubated together for 10 min, the quenched fluorogenic peptide substrate was added to initiate the reaction, and cleavage was monitored by measuring fluorescence increase over time to obtain M^pro^ activity relative to a no inhibitor control. These initial screening experiments identified 4 compounds that appeared to have promising inhibition properties: A02, A08, A22, and A24 (denoted with purple arrows in Figure 3A).

**Figure 3:**
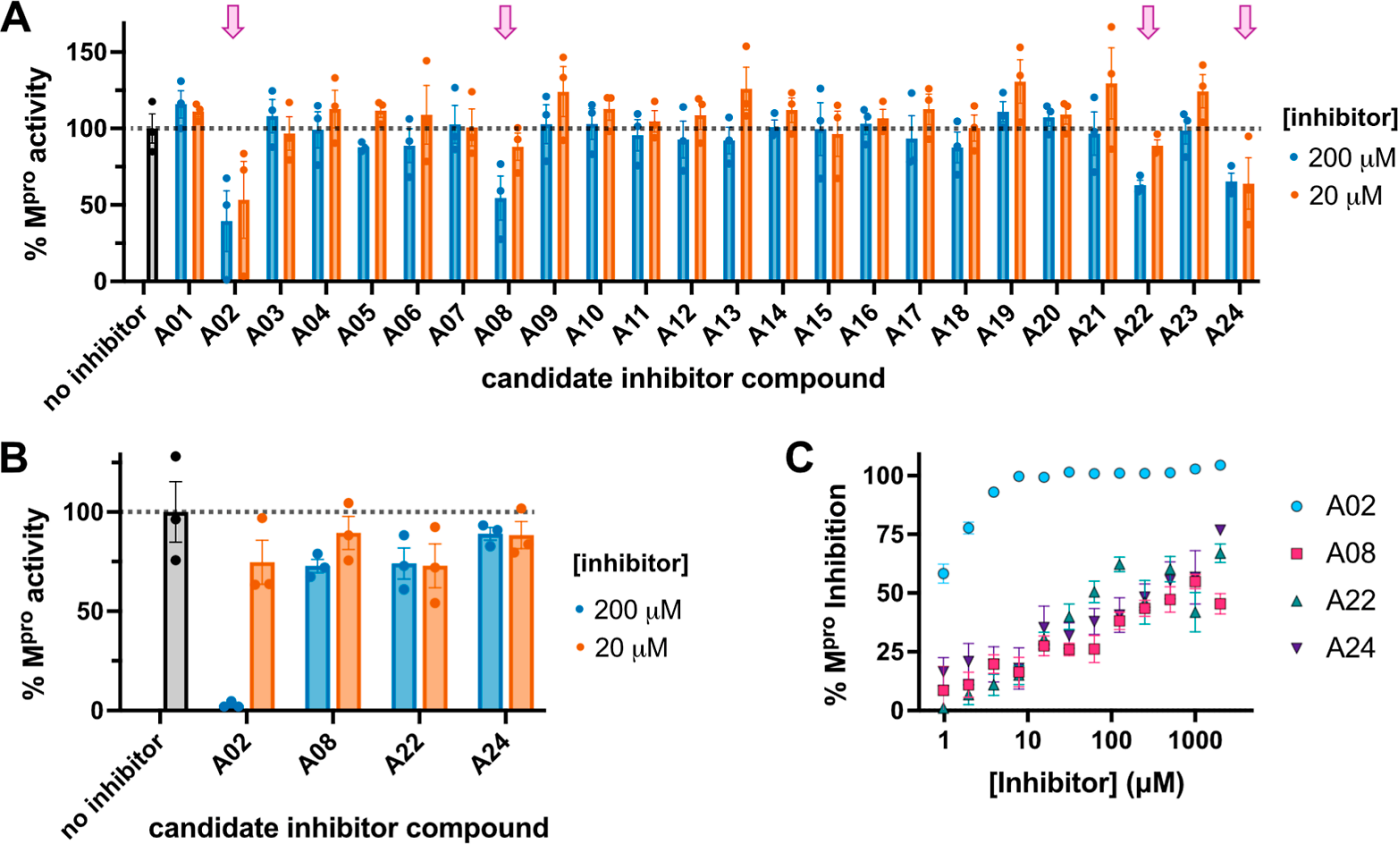
(A) M^pro^ candidate inhibitor screen with compounds A01–A24 selected from computational screening. These assays were carried out with a 10 min inhibitor + M^pro^ preincubation at 20 μM (orange) and 200 μM (blue) final inhibitor concentration. % M^pro^ activity data are shown as mean values ± SEM (*n* = 3), relative to a no inhibitor control (gray bar and dotted line). Purple arrows denote compounds with putative inhibition properties. (B) Focused confirmation screen with top M^pro^ inhibitor candidates selected from (A) (A02, A08, A22, A24). These assays were carried out with a 30 min inhibitor + M^pro^ preincubation at 20 μM (orange) and 200 μM (blue) final inhibitor concentration. % M^pro^ activity data are shown as mean values ± SEM (*n* = 3), relative to a no inhibitor control (gray bar and dotted line). (C) Additional focused confirmation screen with top M^pro^ inhibitor candidates (A02, A08, A22, A24), where the inhibitor + M^pro^ preincubation time was increased to 1 h and final inhibitor concentrations ranging from 1 μM to 2000 μM were tested for each compound.

We next carried out a similar confirmation screen with these 4 most promising candidates (A02, A08, A22, A24), in which M^pro^ and inhibitor were incubated for 30 min prior to the reaction, and again measured percent M^pro^ activity at either 20 or 200 μM inhibitor concentration relative to a no inhibitor control (Figure 3B). Additionally for these 4 compounds, we further increased the M^pro^-inhibitor preincubation time to 1 h and measured percent inhibition of M^pro^ over a wide range of inhibitor concentrations (Figure 3C). In both sets of these inhibition confirmation experiments, we found that compound A02 exhibited robust inhibition of M^pro^, whereas A08, A22, and A24 only weakly inhibited proteolysis activity.

### 2.4 Validation of Candidate A02 as a Covalent M^pro^ Inhibitor

The inhibition assays above suggested that A02 was the most potent inhibitor of M^pro^ activity, but also that this inhibition was time-dependent, where longer preincubation times of A02 with M^pro^ resulted in more potent inhibition activity (comparing A02 data for Figure 3A–C). This is consistent with A02 acting as a covalent inhibitor, which is not unexpected given its chloroacetyl functional group that could react directly with the M^pro^ active site cysteine (Figure 4A). We next carried out measurements to determine the apparent IC50 of A02 for M^pro^ at two different preincubation times, either 1 or 24 h (Figure 4B). The apparent IC50 with 1 h preincubation of M^pro^ and A02 was measured to be 1.5 μM, whereas increasing the preincubation time to 24 h resulted in complete inhibition of M^pro^ at all tested inhibitor concentrations, suggesting a nonmeasurable apparent IC50 ≪ 1.5 μM. These data directly show A02 inhibition of M^pro^ is strongly time-dependent, consistent with covalent inhibition.

**Figure 4:**
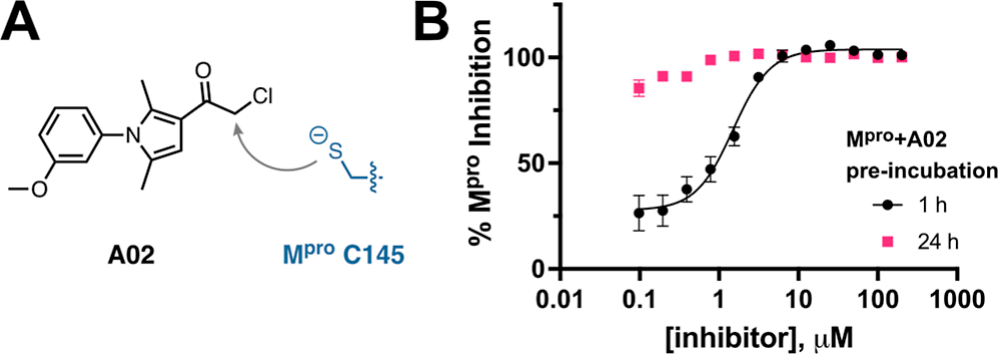
(A) The chloroacetyl group of inhibitor A02 can potentially undergo reaction with M^pro^ C145 to form a covalent adduct. (B) Determination of apparent IC50 for A02 inhibition of 50 nM M^pro^ at two different A02 + M^pro^ preincubation times, 1 h (black) or 24 h (pink). Significantly stronger inhibition is observed for the longer 24 h preincubation, consistent with A02 being a covalent inhibitor.

### 2.5 M^pro^-A02 Crystal Structure

We next determined the structure of inhibitor A02 with M^pro^ to visualize how this compound interacts with the M^pro^ active site (Figure 5 and Table S6). The 1.76 Å resolution structure shows clear density in the *F*_0_–*F*_c_ omit map for A02 covalently bound to the catalytic active site residue C145 in the M^pro^ dimer (Figure 5A). The density for A02 is strongest and most well-defined near the C145-A02 linkage and the A02 pyrrole ring, with somewhat weaker density observed for the A02 methoxyphenyl group, likely due to higher mobility of this ring. An alternative conformation of A02 was also modeled, in which A02 is flipped 180° and linked to C145 in a different side chain orientation (Figure S9). The major A02-C145 conformation pictured in Figure 5 was refined to an occupancy of 77%, with the minor A02-C145 conformation refining to an occupancy of 23%. The A02 methoxyphenyl group occupies the M^pro^ S3′ pocket, packing against the side chains of T25, H41, S46, and M49, and backbone atoms of C44 and T45 (Figure 5B). A02 also makes several hydrogen bond contacts with M^pro^, in which the A02 carbonyl group interacts with the amide NH backbone atoms of G143 and C145, and the methoxyphenyl oxygen atom makes a water-mediated hydrogen bond to M^pro^ (Figures 5C and S10).

**Figure 5:**
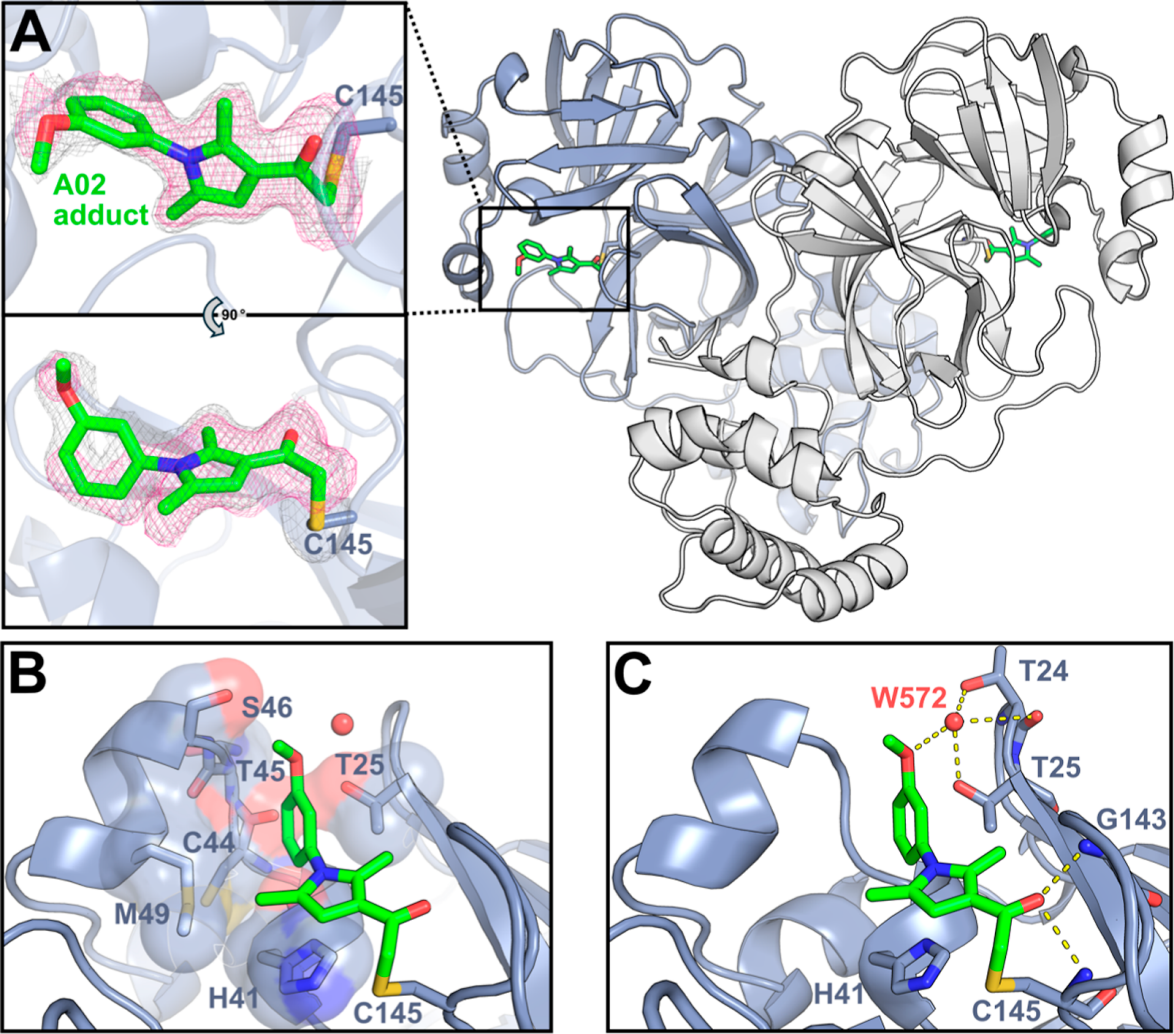
(A) Structure of A02 covalently bound to C145 in the M^pro^ active site. The right panel shows the M^pro^ dimer with each protomer colored differently; A02 (green) is covalently bound to C145 in both active sites of the M^pro^ dimer. The left panels are 90° rotations of the active site A02-C145 adduct showing the *F*_0_–*F*_c_ omit map for the A02 adduct ligand at 2.0 σ (pink mesh) and the refined 2*F*_0_–*F*_c_ map with A02 adduct ligand included at 0.5 σ (gray mesh). (B) The methoxyphenyl group of A02 occupies the M^pro^ S3′ pocket, packing against residues T25, H41, C44, T45, S46, and M49. (C) A02 makes several hydrogen bond contacts in the M^pro^ active site: the A02 carbonyl interacts with the backbone NH groups of G143 and C145; and the A02 methoxyphenyl oxygen interacts with a water molecule (W572, red sphere), which hydrogen bonds to M^pro^ T24 and T25. Hydrogen bond interactions 3.5 Å or under are shown as dashed yellow lines; *F*_0_–*F*_c_ omit map for W572 and hydrogen bonding distances are shown in Figure S10.

### 2.6 A02 as a Novel Covalent Inhibitor Scaffold of SARS-CoV-2 M^pro^

To assess the uniqueness of A02 as a small-molecule inhibitor targeting the M^pro^ S3′ pocket, we performed a structural survey of available M^pro^ crystal structures in the PDB using a distance-based definition of S3′-pocket engagement centered on residues Thr25, Cys44, and Met49 (see Methods). To identify and quantify ligand-S3′ pocket interactions across these structures, we used our recent M^pro^-TRMT1 crystal structure as a reference (PDB 9DW6), since in this structure a Phe side chain from the bound TRMT1 peptide substrate fully occupies the M^pro^ S3′ pocket.^25,26^ We calculated the minimum distance between the peptide Phe benzyl group and M^pro^ S3′ pocket residues Thr25, Cys44, and Met49 in this reference structure as 3.62 Å, 3.61 Å, and 3.49 Å, respectively. Using these reference distances, we defined a minimum distance threshold of 3.7 Å between a ligand and these three S3′ pocket residues as indicative of close contact or binding within the M^pro^ S3′ pocket, which identified only 47 ligands across 52 crystal structures that satisfied these criteria for S3′ pocket binding, out of the 1300+ structures analyzed. Additionally, we conducted a scaffold search among the tested SARS-CoV-2 M^pro^ compounds listed in PubChem and identified only two compounds containing the 1-phenylpyrrole moiety (Figure 6) found in A02. The first, a peptidomimetic covalent inhibitor 5ZB (Figure 6C) included the 1-phenylpyrrole motif but did not exhibit any close contacts with the S3′ pocket (PDB 7RN0). The second, a peptidomimetic noncovalent inhibitor (PubChem CID: 167098835, Figure 6D), also contained the 1-phenylpyrrole motif, but no related crystal structure is available. Notably, both compounds are peptidomimetics that utilize the 1-phenylpyrrole as a side chain and in the opposite orientation to A02. Therefore, this analysis highlights A02 as a novel covalent inhibitor scaffold that uniquely engages the M^pro^ S3′ pocket compared to any previously reported M^pro^ inhibitors.

**Figure 6:**
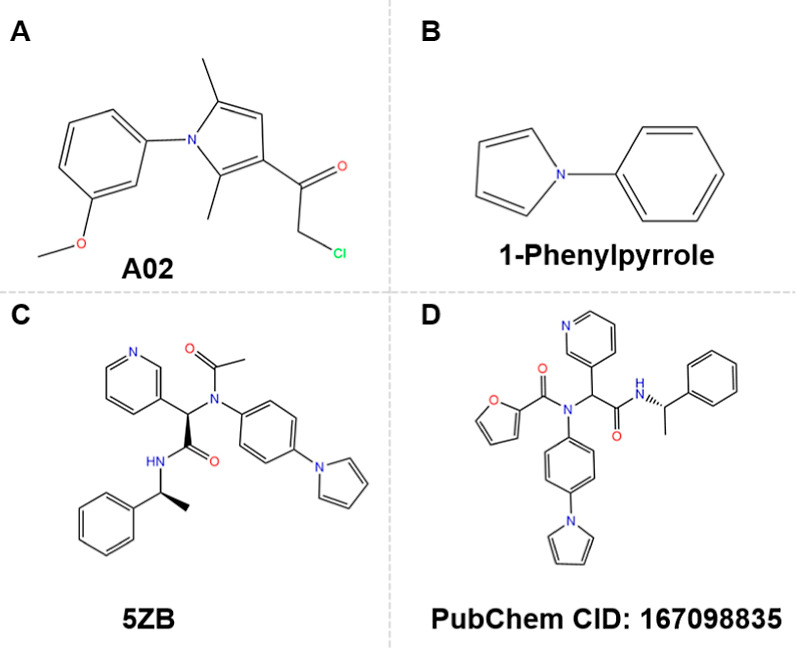
Chemical structures of the A02 scaffold and related M^pro^ inhibitors. (A) The structure of A02. (B) The structure of 1-phenylpyrrole. (C) The structure of the peptidomimetic covalent inhibitor 5ZB. (D) The structure of the noncovalent inhibitor (PubChem CID: 167098835).

### 2.7 AlphaSpace2 Pocket Analysis Uncovers Optimization Potential for A02

Next, to assess the potential of covalent inhibitor A02 as a starting point for further chemical elaboration and rational drug design, pocket analysis using AlphaSpace2 ^27^ was conducted to evaluate the binding characteristics of A02 and adjacent M^pro^ binding site pockets. Figure 7 summarizes the AlphaSpace2 analysis, highlighting nearby vacant M^pro^ binding pockets in different colors and showing numbered spheres at the optimal positions for a ligand moiety to occupy each AlphaSpace2-defined pocket. The Bscore parameter listed in the associated table in Figure 7 provides an estimate of pocket ligandability, and the Space parameter indicates the volume of the empty pocket; large negative Bscores and larger pocket volumes suggest the most favorable sites for additional binding interactions. A02 predominantly interacts with the S3′ site (pocket #1), indicating this is a critical region for stabilizing binding through hydrogen bonding or hydrophobic interactions with A02. However, the high-scoring pockets #3 and #4 directly adjacent to A02 in the M^pro^ S3 and S1 sites appear to be underutilized by A02 with calculated occupancies of only 35.4% and 12.21%, respectively. These results suggest that A02 has potential to serve as a starting scaffold for future derivatization. In particular, the adjacent pockets identified by AlphaSpace2 may provide opportunities for extending the A02 scaffold to form additional interactions with M^pro^. However, we have not yet synthesized or experimentally evaluated such derivatives, and therefore this analysis should be interpreted as identifying structure-guided opportunities for future optimization rather than demonstrating that optimization has already been achieved (see Fig. 8).

**Figure 7:**
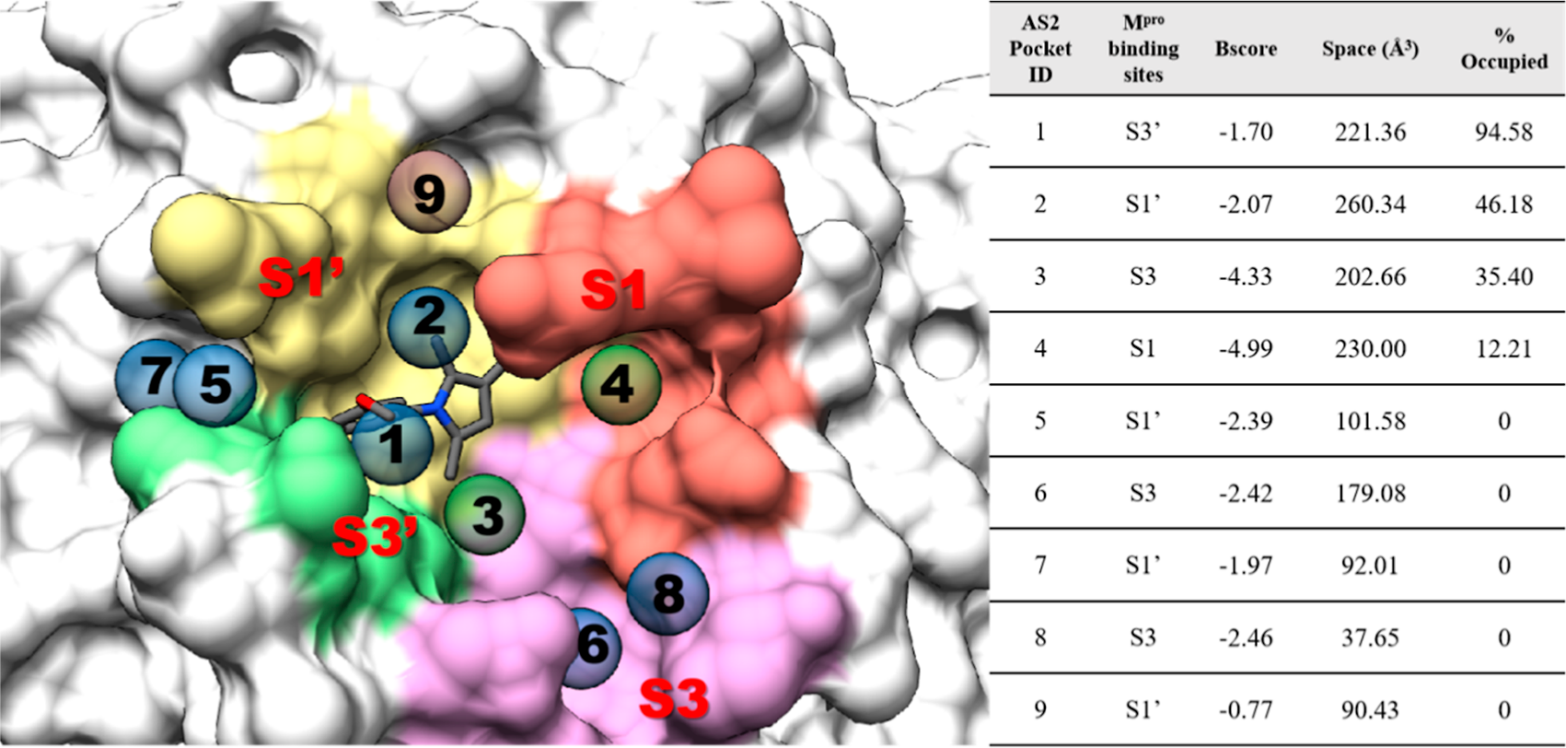
3D Mapping of A02 binding and pocket occupancy across the M^pro^ active site using AlphaSpace2 (left). The AlphaSpace2 Pocket IDs, corresponding M^pro^ binding sites, Bscores, Space parameter, and % Occupancy are listed in companion table (right). High (Bscore < −2.5 kcal/mol), mid (−2.5 kcal/mol ≥ Bscore < −1.5 kcal/mol), and low (Bscore ≥ −1.5 kcal/mol) scoring pockets are colored with green, blue, or brown spheres, respectively. The M^pro^ active site pockets are highlighted in different colors. This figure shows AlphaSpace2 analysis for A02 in the major binding conformation, as shown in Figure 5; AlphaSpace2 analysis for A02 in the alternative/minor binding conformation, as shown in Figure S9, is shown in Figure S11.

**Figure 8:**
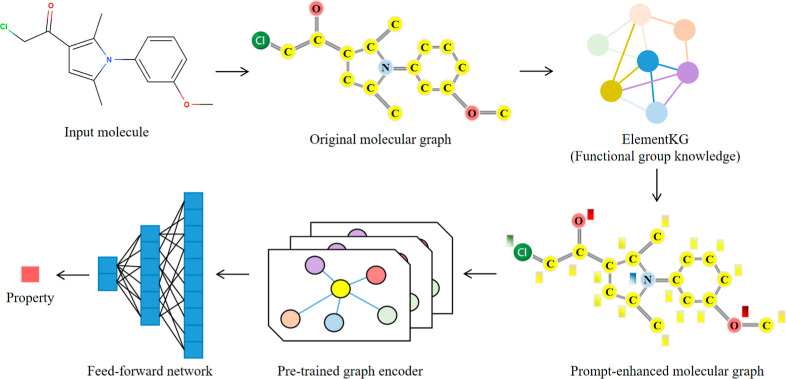
Simplified Overview of KANO. The input molecule is first converted into its original molecular graph. Next, the functional groups and elements within the molecule are detected, and their corresponding entity embeddings are retrieved from ElementKG. These functional embeddings are then incorporated into the original molecular graph, resulting in a prompt-enhanced molecular graph. This new molecular graph is subsequently used to fine-tune the pretrained graph encoder, enabling it to perform downstream molecular property prediction tasks, including logS, logP, and M^pro^-inhibitor activity prediction.

## 3 Conclusion

In this work, we used a deep learning-based workflow for identifying potential inhibitors against the SARS-CoV-2 M^pro^. The computational workflow included a target-specific M^pro^-inhibitor model, which achieved a precision of 0.883 on the test set, demonstrating a high true positive rate. Additionally, logS and logP models were used to evaluate the drug-likeness of the candidates, both showing strong performance in their respective property prediction tasks. Molecular similarity calculations and literature searches were also conducted to ensure the novelty of the drug candidates. Applying this protocol to the Asinex chemical library, 24 compounds were selected for bioassay testing, leading to the identification of one novel covalent inhibitor.

The compound A02 was one of the highest scoring computationally predicted candidate inhibitors, and in in vitro M^pro^ inhibition assays we found that this molecule potently inhibited protease activity on a model substrate in a time-dependent manner. The experimentally determined apparent IC_50_ for A02 with M^pro^ was 1.5 μM with a 1 h preincubation of M^pro^ and A02, whereas increasing the preincubation time to 24 h resulted in a substantial increase in inhibition and dramatic decrease in the apparent IC50 that was too low to be accurately quantified in this assay. We next determined the structure of A02 bound to M^pro^, revealing that the chloroacetyl group of A02 reacts with the catalytic active site residue C145 to form a covalent adduct. A02 also occupies the M^pro^ S3′ pocket and makes several hydrogen bond contacts with additional M^pro^ residues. The S3′ pocket is relatively under-utilized by existing M^pro^ inhibitors, and as our AlphaSpace analysis shows, functionalization of the A02 pyrrole ring has the potential to introduce further groups that engage adjacent M^pro^ surfaces and pockets for enhanced affinity, selectivity, and inhibition properties. A02 thus represents a novel M^pro^ inhibitor scaffold obtained from our virtual screening workflow and provides a promising starting point for future structure-guided optimization.

Overall, this work links target-specific deep learning prioritization to prospective biochemical testing and high-resolution structural validation. From a purchasable library of 575,302 compounds, the workflow selected 24 candidates for testing and identified A02, a covalent inhibitor fragment with low-micromolar apparent potency and a 1.76 Å M^pro^ complex structure that reveals a distinctive binding mode engaging the S3′ pocket. Together with the pocket-occupancy and AlphaSpace analyses, these results provide a concrete starting point for scaffold derivatization aimed at improving affinity and selectivity through additional interactions with adjacent M^pro^ surfaces and pockets.

## 4 Materials and Methods

### 4.1 Data Collection

In this work, we collected three data sets to perform various molecular property prediction tasks: the M^pro^-inhibitor data set, the aqueous solubility (logS) data set, and the logP data set. These data sets are summarized in Table 1.

**Table 1:** Summary of the Datasets

data set	total no. of records	source(s)
M^pro^-inhibitor data set	13,484 (active: inactive^a^ = 1645:11,839)	● NIH assay^18^
		● moonshot^19^
		● literature
LogS data set	4428 (cleaned)	● AQUA
		● ESOL
		● PHYS
LogP data set	14,176	● PHYSPROP

a Active compounds are defined as those with an IC50 value of less than 10 μM or an inhibition level below −50% in the NIH assay. Inactive compounds are defined as those with an IC50 value of 10 μM or greater, or an inhibition level of −50% or higher in the NIH assay.

The M^pro^-inhibitor data set was assembled from the NIH 3CL assay, COVID Moonshot, and published literature sources.^28–69^ After collection, the data set was cleaned by standardizing compound records, removing invalid or duplicate entries, and harmonizing activity annotations across sources prior to temporal splitting. A detailed description of the M^pro^ data set cleaning workflow is provided in the Supporting Information (Figure S1 and related text). After data cleaning, a temporal split was applied, with pre-2021 data used for training and post-2021 data used for testing. We selected 2021 as the cutoff because it marks a practical transition between the initial wave of SARS-CoV-2 M^pro^ inhibitor data generated in 2020 and the broader body of compounds reported thereafter. This design was intended to approximate a prospective screening scenario, in which a model trained on earlier available data is evaluated on compounds disclosed at later stages of the pandemic, and therefore provides a more stringent and realistic assessment of model generalizability than a conventional random split (see Figure S1). For the logS model, duplicate and overlapping entries among the AQUA (1311 compounds), ESOL (1128 compounds), and PHYS (2010 compounds) data sets were averaged for model development. To assess generalizability, overlapping records were used as the training/validation set, while the unique records were used as an external test set. This test set contained 1204 compounds in total, comprising 182 AQUA compounds, 183 ESOL compounds, and 839 PHYS compounds.^20–22^ In addition, we collected 135 orally available drugs with experimentally determined logS and logP values from DrugBank to examine the joint distribution of these properties and define the screening range. Because some of these 135 drugs overlapped with the logS data set used for model development, overlapping compounds were excluded from the independent validation analysis, leaving 62 nonoverlapping approved drugs for the validation shown in Figure 1D. Data cleaning is described in the Supporting Information. The logP data set comprised 14,176 compounds from PHYSPROP, focused on lipophilicity.^23^ Lastly, the Asinex virtual library, with over 575,000 compounds, was screened using KANO fine-tuned models for rapid analysis.

### 4.2 Model and Training

A recently proposed deep learning method, knowledge graph-enhanced molecular contrastive learning with functional prompt (KANO),^15^ consists of three main components: (1) ElementKG construction and embedding, (2) contrastive-based pretraining and (3) prompt-enhanced fine-tuning. The key component of KANO is the ElementKG (chemical element-oriented knowledge graph), which combines the basic knowledge of elements and functional groups. The simplifed overview of KANO is shown in Figure 8. This deep learning model has previously demonstrated strong performance across a range of molecular property prediction tasks.^15^ In this work, we fine-tuned KANO for specific property prediction tasks, including M^pro^-inhibitor activity prediction, aqueous solubility (logS) prediction, and logP prediction.

The hyperparameters are listed in Table S2.

The KANO-M^pro^ inhibitor model aimed to address class imbalance by testing three training strategies: maintaining the original distribution, undersampling inactive compounds, and oversampling active compounds. Each approach was evaluated to mitigate bias while preserving performance. For the KANO-logS model, logS values from overlapping entries in the AQUA, ESOL, and PHYS data sets were averaged, and two different data splits were explored to improve model generalizability. The KANO-logP model utilized a random split of the PHYSPROP data set across five seeds, with ensemble predictions enhancing overall model accuracy. Details of the model training and evaluation metrics are provided in the Supporting Information.

To evaluate the prediction performance of the classification model, accuracy, recall, precision, and the F1-score were used as statistic metrics, as calculated by equations 1–4):

1 $$Accuracy=\frac{Number\;of\;correct\;predicitions}{Total\;number\;of\;predicitons}$$

2 $$Precision=\frac{True\;Positives}{True\;Positives+False\;Positives}$$

3 $$Recall=\frac{True\;Positives}{True\;Positives+False\;Negatives}$$

4 $$F1‐score=2\times \frac{Precision\times Recall}{Precision+Recall}$$

For the prediction performance of the regression model, RMSE (root-mean-square deviation), *R* ^2^ (coefficient of determination), pearson correlation coefficient (PCC), and mean absolute error (MAE) were used as statistic metrics, as calculated by eq 5–8):

5 $$RMSE=\sqrt{\frac{\sum_{i=1}^{n}{\left(y_{i}-{ŷ}_{i}\right)}^{2}}{N}}$$

6 $$R^{2}=1-\frac{\sum_{i=1}^{n}{\left(y_{i}-{ŷ}_{i}\right)}^{2}}{\sum_{i=1}^{n}{\left(y_{i}-{ŷ}_{i}\right)}^{2}}$$

7 $$r=\frac{\sum_{i=1}^{n}\left(y_{i}-{\mathrm{y̅}}_{i}\right)\left({ŷ}_{i}-{\overset{®}{\hat{y}}}_{i}\right)}{\sqrt{\sum_{i=1}^{n}{\left(y_{i}-{\mathrm{y̅}}_{i}\right)}^{2}\sum_{i=1}^{n}{\left({ŷ}_{i}-{\overset{®}{\hat{y}}}_{i}\right)}^{2}}}$$

8 $$MAE=\frac{1}{n}\sum_{i=1}^{n}\left|\hat{y_{i}\;}-y_{i}\right|$$

where *y*_*i*_ and ${ŷ}_{i}$ represent the reported value and the predicted value of sample *i*, respectively, ${\mathrm{y̅}}_{i}$ is the average of *y*_*i*_, ${\overset{®}{\hat{y}}}_{i}$ is the average of ${ŷ}_{i}$ , and *n* is the number of samples.

### 4.3 Deep Learning-Based Virtual Screening Workflow

In our workflow, molecular similarity is the final step to assess novelty by comparing drug candidates to our M^pro^-inhibitor data set and PubChem compounds (19,677 records).^70^ Molecular similarity quantifies the likeness between molecules using descriptors or fingerprints, with metrics such as the Tanimoto coefficient (*T*_c_) or Dice coefficient (*D*_c_).^71^ In cheminformatics, the Morgan fingerprint is widely used for this purpose.^72^ Similarity is calculated using RDKit Morgan fingerprint bit-vectors, and candidates with *T*_c_ > 0.85 are excluded.^73^

We collected approved drug data from DrugBank^74^ and analyzed their logS and logP values to identify an optimal range for these properties. We found 135 orally available drugs with experimentally determined logS and logP (Figure S2). Based on the distribution of logS and logP, we determined that a drug candidate with a logS between −5 and 0 and a logP between 0 and 5 tends to be orally available. This range was then used as a benchmark to evaluate whether potential drug candidates fell within the desired range, indicating their potential suitability as viable drugs.

The models were applied to the Asinex Virtual Library, and positive predictions from the M^pro^-inhibitor model underwent a druglikeness evaluation. Compounds were filtered based on predicted logS (−5 to 0), predicted logP (0 to 5), and a Tanimoto coefficient (*T*_c_ < 0.85) to ensure suitable solubility, lipophilicity, and novelty. Following this, an extensive literature search in databases such as PubChem and ChEMBL^75^ confirmed that these compounds had not been previously tested for M^pro^-inhibitor activity. The top 24 compounds with the highest predicted probability of being active inhibitors were selected for further bioassay testing.

### 4.4 M^pro^ Expression and Purification

The wild-type M^pro^ enzyme (catalog #141370, Addgene) was purified as described in our previous work.^10^ In summary, a human codon-optimized M^pro^ gene was expressed with an N-terminal GST tag followed by an AVLQ autocleavage site to yield a native M^pro^ N-terminus after self-cleavage. The expression plasmid was transformed into *E. coli* Rosetta­(DE3)­pLysS cells, and colonies were selected on LB agar containing 50 μg/mL ampicillin. Starter cultures were grown overnight in LB media supplemented with 50 μg/mL ampicillin at 37 °C with shaking at 200 rpm. A 1:100 dilution of the starter culture was inoculated into 1 L of fresh LB media and incubated at 37 °C until the OD600 reached 0.6. Protein expression was induced with 1 mM IPTG, and cultures were then shifted to 18 °C for overnight incubation with shaking. Cells were collected by centrifugation at 7,500*g*, and the pellet was stored for lysis. Lysis was performed by sonication in buffer containing 25 mM *Tris* (pH 8.0), 300 mM NaCl, and 10 mM imidazole, followed by centrifugation at 14,500*g* for 45 min. The resulting clarified lysate was incubated with 1.5 mL of equilibrated HisPur Ni-NTA resin for 30 min with gentle mixing. The resin was added into a gravity flow column and washed sequentially with 25 mL of lysis buffer and then 25 mL of lysis buffer with 25 mM imidazole. The target protein was eluted using 10 mL of lysis buffer containing 250 mM imidazole. The eluted protein was concentrated to about 2 mL using a 10 kDa MWCO centrifugal concentrator and subjected to size exclusion chromatography on a HiLoad 16/600 Superdex 200 pg column. Purified M^pro^ fractions were concentrated to 10–25 mg mL^–1^, flash-frozen in liquid nitrogen, and stored at −70 °C in a buffer of 50 mM *Tris* (pH 7.3), 1 mM EDTA, and 2 mM DTT.

### 4.5 M^pro^ Inhibition Assays

Initial screens for M^pro^ inhibition by candidate compounds A01–A24 (shown in Figure S8) were carried out in Corning Low Volume 384-well Black Flat Bottom Polystyrene NBS Microplates. Reactions were set up in triplicate with 200 nM (final concentration) WT M^pro^ enzyme in a buffer containing 50 mM Tris (pH 7.3), 1 mM EDTA, 2 mM DTT, and 20% DMSO. The inhibition assay utilized a quenched fluorescent peptide substrate at an optimized final concentration of 25 μM. The synthetic peptide substrate, the nsp4/5 cleavage sequence (MCA-SAVLQSGFRKM-K­(Dnp)­K), was sourced from Peptide 2.0. Each of the 24 small-molecule inhibitors were initially tested at two final concentrations (200 μM and 20 μM), after preincubation at 2× concentration with 2× M^pro^ for 10 min at RT. M^pro^-only controls were preincubated with buffer and the equivalent amount of DMSO present in the compound dilution series at each concentration. Following preincubation, 2× fluorescent peptide substrate was added to initiate proteolysis reactions at 1× final concentrations, and the fluorescence (excitation: 320 nm, emission: 405 nm) was monitored every 10 s for 3 min using a Tecan Spark microplate reader. To convert relative fluorescence units (RFU) to μM peptide cleavage product, a calibration curve was generated with synthetic product MCA-AVLQ ranging from 12 to 0.006 μM. The inner filter effect (IFE) correction was calculated as follows: IFEcorr = [fluorescence_MCA_product+peptide_ – fluorescence_peptide_]/fluorescence_MCA,_ where fluorescence_MCA_product+peptide_ represents the fluorescence of MCA-AVLQ mixed with the peptide substrate, fluorescence_peptide_ is the fluorescence of the peptide alone, and fluorescence_MCA_ is the signal of the MCA-AVLQ product. Initial reaction rates (μM/s) were compared directly to the M^pro^-only control to determine percent activity or percent inhibition relative to the uninhibited M^pro^ cleavage reaction.

For the focused confirmation screens in Figure 3B,C, the assays were conducted as above, with a longer preincubation of M^pro^ + compound for 30 min or 1 h at RT. To determine the IC_50_ of A02, M^pro^ (50 nM final concentration) and A02 dilution series (200–0.098 μM final concentrations) were mixed and incubated for 1 or 24 h before initiating the reaction with fluorogenic peptide substrate. Plots of % M^pro^ inhibition (relative to a no inhibitor control) versus A02 concentration were fit to the “[Inhibitor] vs response—Variable slope (four parameters)” model in GraphPad Prism 10 to obtain the apparent IC_50_. We refer to the fitted IC_50_ value here as an “apparent IC_50_”, because A02 is a covalent (nonreversible) inhibitor whose IC_50_ is time-dependent.

### 4.6 M^pro^-A02 Crystallization

Ten mg mL^–1^ purified M^pro^ was preincubated with 1 mM A02 for 2 h at RT. The protein-compound mixture was then diluted 1:1 with optimized precipitant solution (26% PEG 6000, 100 mM HEPES, pH 7.5), and set as a 2 μL hanging drop in 24-well VDX plates with 500 μL precipitant solution in the well. Single rhombohedral crystals were grown at 20 °C and harvested with 20% glycerol added as a cryoprotectant during looping. Crystals were flash frozen in liquid nitrogen.

### 4.7 M^pro^-A02 Structure Solution and Refinement

Diffraction data were collected at the National Synchrotron Light Source II (NSLS II) highly automated macromolecular crystallography (AMX) beamline 17-ID-1 at the Brookhaven National Laboratory on an Eiger 9 M Pixel detector at 100 K and a wavelength of 0.920105 Å. Diffraction data were indexed, integrated, and scaled using autoproc. The M^pro^-A02 structure was solved in space group C2 using the Phaser package in the CCP4 suite and a modified PDB 7BB2 as search model. After initial rounds of refinement in PHENIX to model M^pro^ residues, A02 was manually built into the *F*_o_–*F*_c_ map using COOT, with initial restraints and starting ligand geometry from PHENIX eLBOW. Subsequent rounds of automated refinement and water placement using PHENIX, updated restraints for A02 obtained from grade2 web server, and manual adjustments including modeling an alternative A02-C145 conformation in COOT were used to obtain the final structure.

### 4.8 Structural Survey of M^pro^ S3′-Pocket Occupancy

To assess the uniqueness of A02 as a small-molecule inhibitor occupying the M^pro^ S3′ pocket, we performed a structural survey of available M^pro^ crystal structures in the Protein Data Bank (PDB). Based on the previous structural analysis, the S3′ pocket was represented by three key residues: Thr25, Cys44, and Met49.^25^ A total of 1746 SARS-CoV-1 and SARS-CoV-2 M^pro^ PDB files were collected from combined in-house collections and keyword-based searches. Structures were retained for analysis only if at least one protein chain contained all three key residues with the correct residue identities and numbering; 1347 PDB files satisfied this criterion.

Only nonwater HETATM records were considered as potential ligands. If a ligand was composed of multiple disconnected parts with different residue names, each part was analyzed separately as an independent ligand. For each ligand, the minimum distance to Thr25, Cys44, and Met49 was calculated. As a reference for S3′-pocket occupancy, we used the M^pro^–TRMT1 complex structure (PDB 9DW6), in which a P3′ phenylalanine side chain occupies the S3′ pocket. In this reference structure, the minimum distances from the phenyl side chain to Thr25, Cys44, and Met49 were 3.62, 3.61, and 3.49 Å, respectively. Based on these values, we used a stringent cutoff of 3.7 Å for each of the three residue distances to define close S3′-pocket engagement. Using this criterion, 47 ligands across 52 crystal structures were identified as occupying the S3′ pocket.

A broader distance-sum analysis was also performed during initial filtering, but the stricter per-residue cutoff described above was used for the final structural comparison reported in Section 2.6.

## Supplementary Material



## Data Availability

The source code and data sets used in this study, including the processed data sets used for model training and evaluation, are publicly available at the GitHub repository: https://github.com/WeiJunZhou23/Mpro-inhibition_project.

## Notes

Supplementary Figure 1: Summary of the M^pro^-inhibitor data cleaning and time-based data set splitting; Supplementary Figure 2: Distribution of logS and logP for 135 orally available drugs; Supplementary Figure 3: Distribution of logP (OpenChem) for 14176 compounds; Supplementary Figure 4: Distribution of logS of AQUA, ESOL, and PHYS data sets; Supplementary Figure 5: Overlapping Between AQUA, ESOL, and PHYS; Supplementary Figure 6: LogS Model Performance Comparison; Supplementary Figure 7: LogS model performance on external data; Supplementary Figure 8: Chemical structures of the top 24 drug candidates; Supplementary Figure 9: Crystallographic modeling of the covalent A02–M^pro^ C145 adduct; Supplementary Figure 10: Detailed view of key interactions between A02 and M^pro^; Supplementary Figure 11: Three-dimensional mapping of A02 binding across Mpro binding sites for the alternate ligand conformation, including AlphaSpace2 pocket assignments, binding scores, and site occupancies; Supplementary Table 1: Summary of record for the AQUA, ESOL, and PHYS logS data sets; Supplementary Table 2: Hyperparameter settings used for training KANO models; Supplementary Table 3: Test-set performance of the KANO M^pro^-inhibitor classification model; Supplementary Table 4: Comparison of logP prediction performance across multiple models on the OpenChem data set; Supplementary Table 5: Predicted inhibition probability, logP, logS, and chemical similarity (Tanimoto coefficient) for the 24 top-ranked drug candidates suggested by the model; Supplementary Table 6: Crystallographic data collection and refinement statistics for the A02–M^pro^ complex (PDB ID: 9E9P). M^pro^-inhibitor data set collection details, aqueous solubility (logS) data set cleaning details, and three KANO finetuned models’ training details are provided (PDF)
