# Supplementary material for: A Novel Covalent Inhibitor Fragment for the SARS-CoV‑2 Main Protease Identified by Target-Specific Deep Learning
Source: ACS Chem Biol. 2026 May 1;21(5):1112–24. doi: 10.1021/acschembio.6c00120 (PMC13184936; doi:10.1021/acschembio.6c00120)
Supplement: Supplementary file 1 [file cb6c00120_si_001.pdf]

Supporting Information

A Novel Covalent Inhibitor Fragment for the SARS-CoV-2 Main Protease Identified by Target-Specific Deep Learning

Weijun Zhou[1*], Angel D′Oliviera[2*], Xuhang Dai[1], Jeffrey S. Mugridge[‡2], Yingkai Zhang[‡1,3,4]

1. Department of Chemistry, New York University, New York, New York 10003, United States

2. Department of Chemistry & Biochemistry, University of Delaware, Newark, DE 19716

3. Simons Center for Computational Physical Chemistry at New York University, New York, New York 10003, United States

4. NYU-ECNU Center for Computational Chemistry at NYU Shanghai, Shanghai 200062, China

[*]Signifies authors with equal contribution

[‡]Signifies corresponding authors

Supplementary Figures

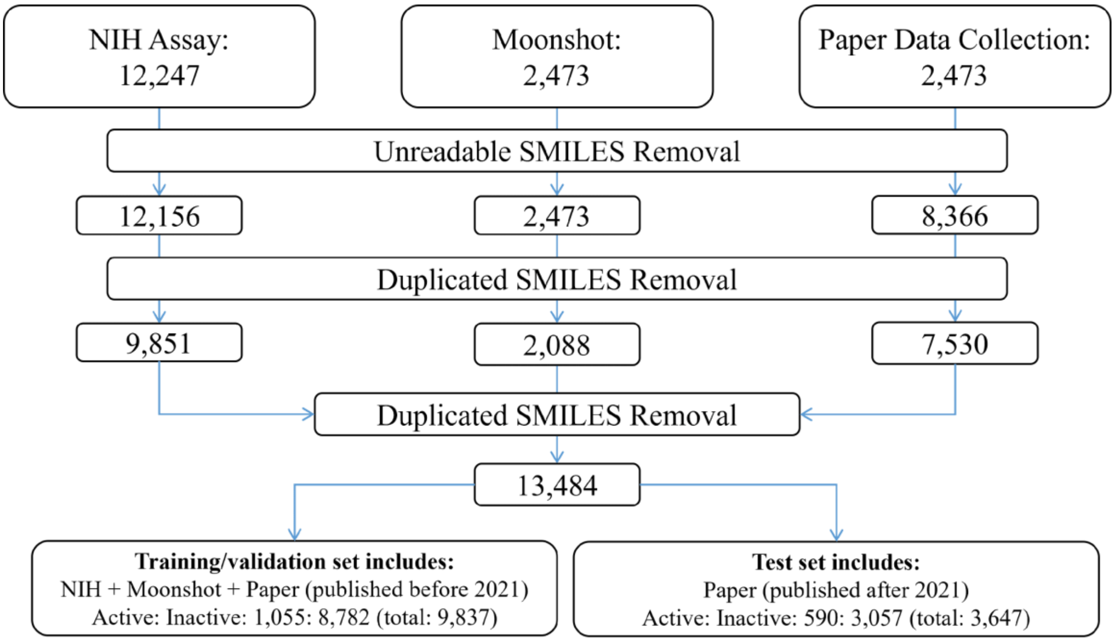

Supplementary Figure 1. Summary of the M^{pro}-inhibitor data cleaning and time-based dataset splitting.

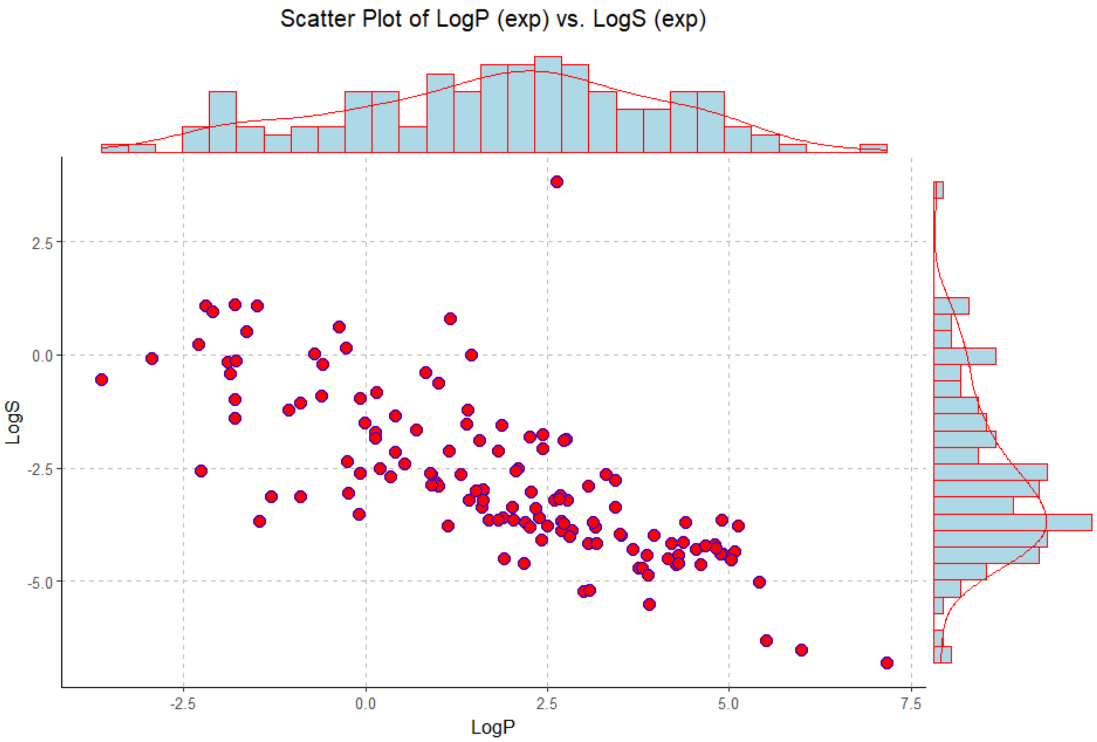

Supplementary Figure 2. Distribution of logS (y-axis) and logP (x-axis) for 135 orally available drugs.

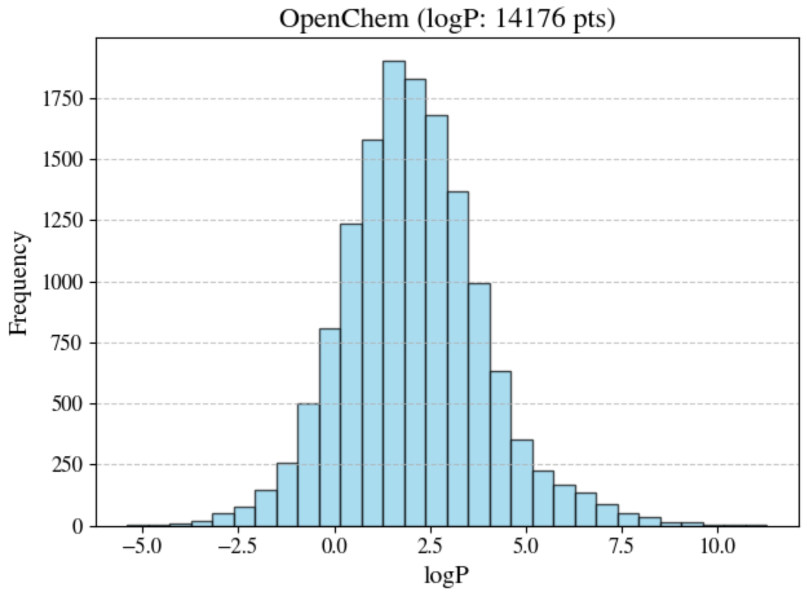

Supplementary Figure 3. Distribution of logP (OpenChem) for 14176 compounds.

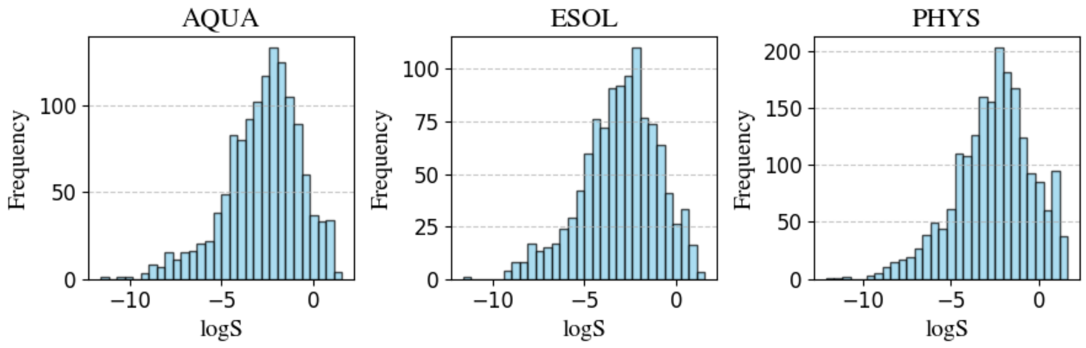

Supplementary Figure 4. Distribution of logS of AQUA, ESOL, and PHYS datasets.

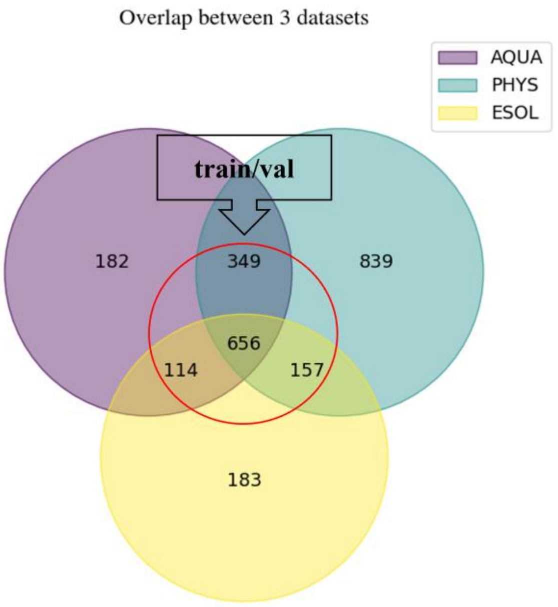

Supplementary Figure 5. Overlap among the AQUA (light purple), ESOL (light yellow), and PHYS (mint green) logS datasets. In the corresponding fixed-split logS analysis, overlapping molecules were used for training/validation and unique molecules were used as the test set.

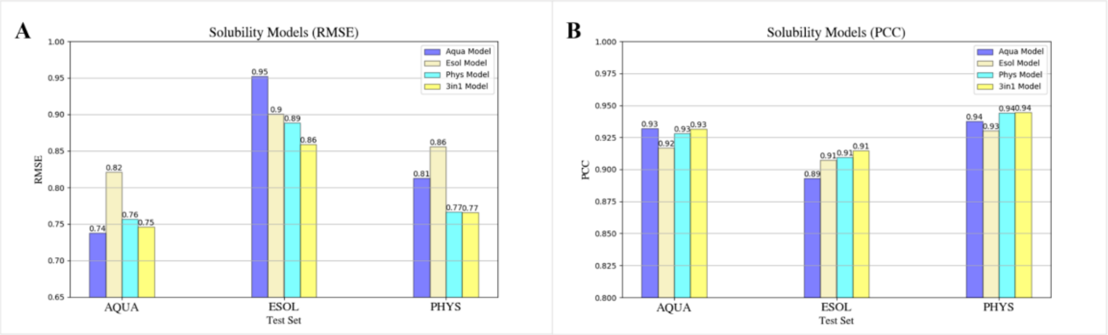

Supplementary Figure 6. LogS Model Performance Comparison: *A*. RMSE of four models tested on the AQUA, ESOL, and PHYS datasets. *B*. PCC of the same models on these test sets. Each test set contains a different number of samples (AQUA: 182, ESOL: 183, PHYS: 839). The models were trained on corresponding training/validation sets as follows: AQUA model (1,119 samples), ESOL model (927 samples), PHYS model (1,162 samples), and a combined "3in1" model using all datasets (1,276 samples). These test sets correspond to the datasets described in Figure 2.

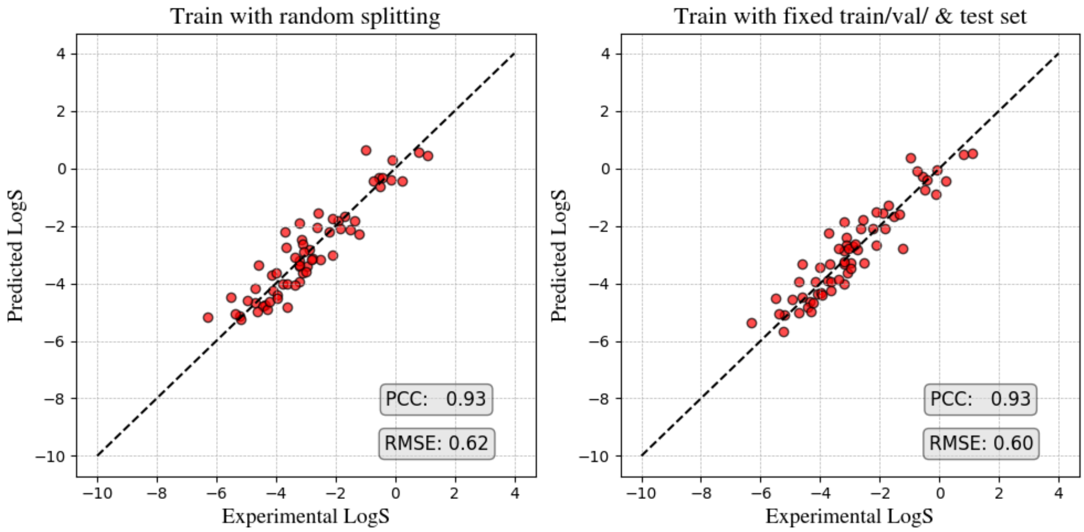

Supplementary Figure 7. Predicted versus experimental aqueous solubility (logS) for 62 approved drugs from DrugBank. Models trained using random splitting (left) or fixed training/validation and test sets (right) show comparable performance. PCC and RMSE values are reported.

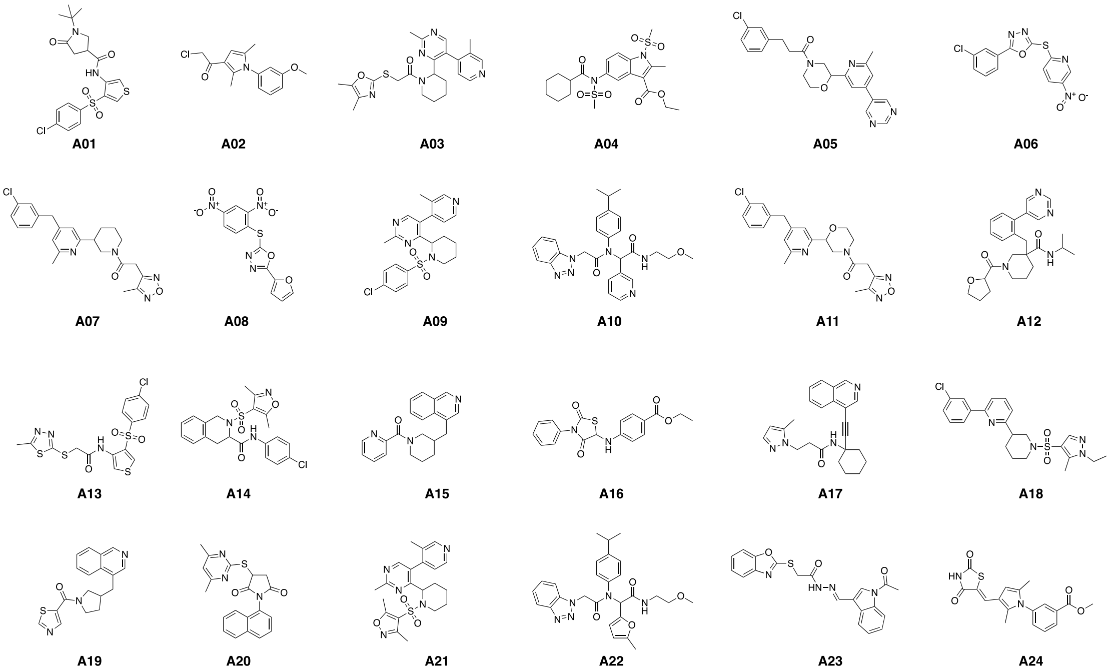

Supplementary Figure 8. Chemical structures of the top 24 drug candidates.

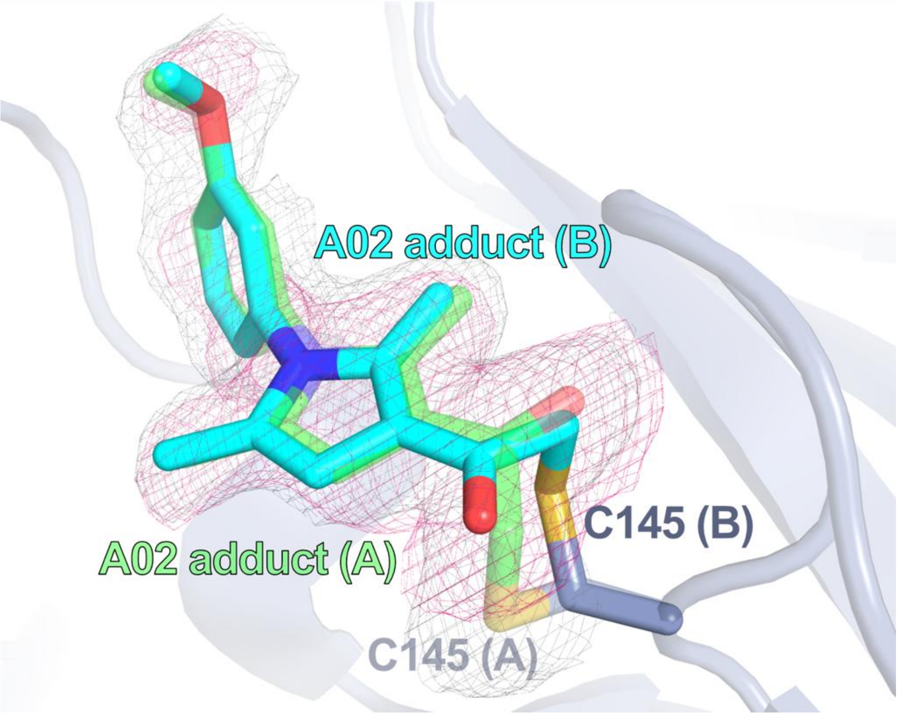

Supplementary Figure 9. The covalent adduct of A02 to M^{pro} C145 was modeled as two alternative conformations (A and B above), where A02 is flipped 180 degrees and bound to C145 in an alternative sidechain orientation. The major conformation is A (refined to 77% occupancy; shown as transparent sticks with green A02), and the minor conformation is B (refined to 23% occupancy; shown as solid sticks with cyan A02). The major alternative conformation A is shown in all the figures in the main text. The F_o-F_c omit map for the A02 adduct ligand at 2.0 σ (pink mesh) and the refined $2F_o$-F_c map with A02 adduct ligand included at 0.5 σ (gray mesh) are shown.

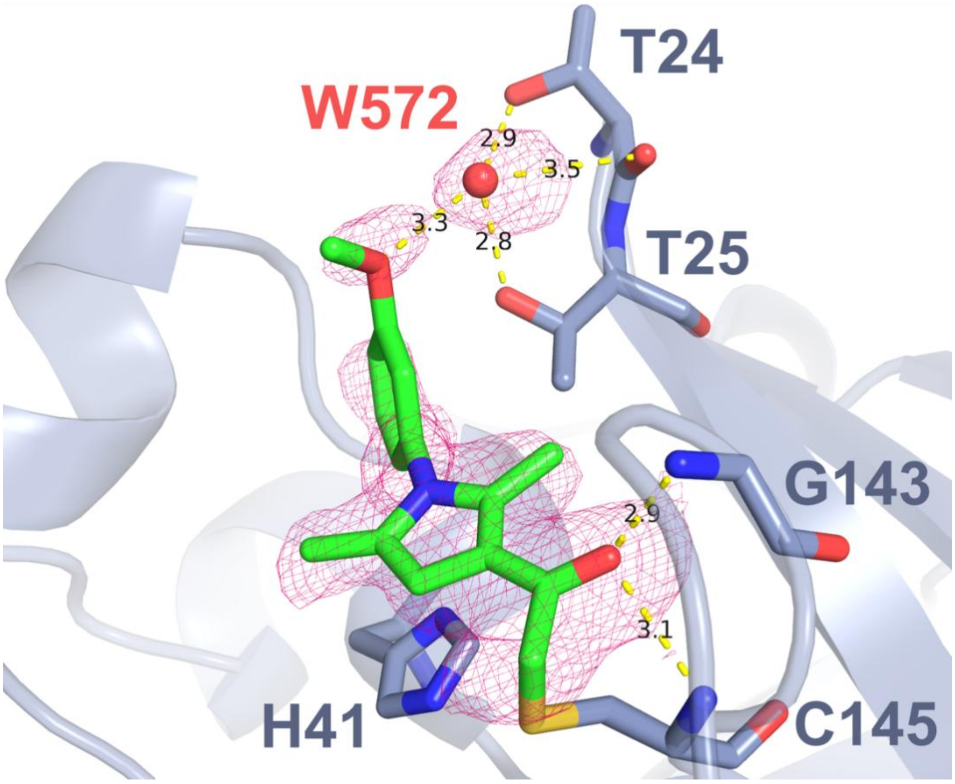

Supplementary Figure 10. The A02 carbonyl group makes hydrogen bond contacts with NH backbone atoms of M^{pro} G143 and C145; the A02 methoxyphenyl oxygen atom makes a water-mediated (W572) hydrogen bonding interaction with M^{pro} T24 and T25. The F_o-F_c omit map for A02 and W572 is shown at 2.0 σ (pink mesh); hydrogen bonding interactions are shown as yellow dashed lines with distances in Angstroms.

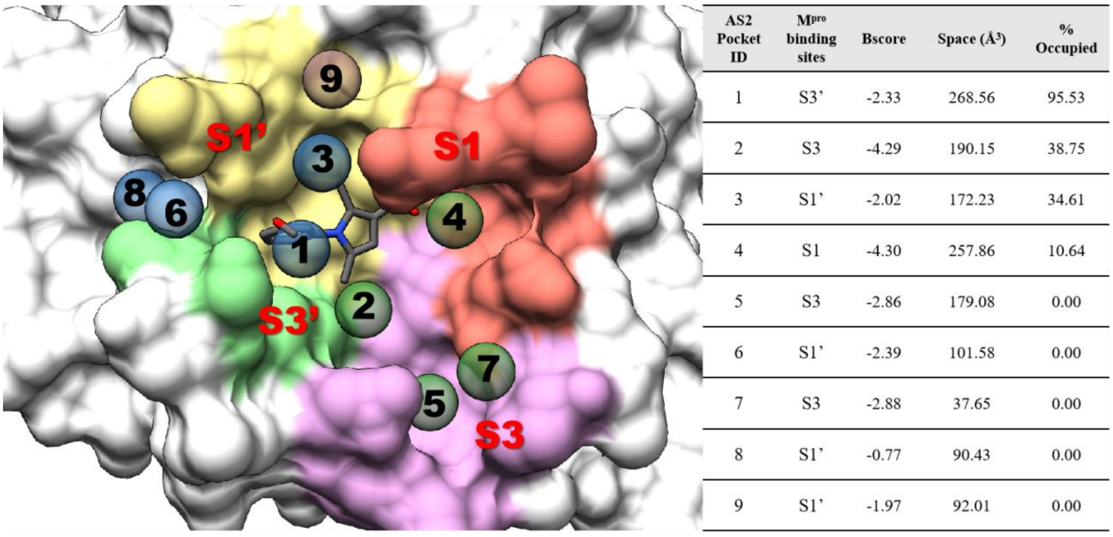

AS2 Pocket ID	M^{pro} binding sites	Bscore	Space (Å³)	% Occupied
1	S3'	-2.33	268.56	95.53
2	S3	-4.29	190.15	38.75
3	S1'	-2.02	172.23	34.61
4	S1	-4.30	257.86	10.64
5	S3	-2.86	179.08	0.00
6	S1'	-2.39	101.58	0.00
7	S3	-2.88	37.65	0.00
8	S1'	-0.77	90.43	0.00
9	S1'	-1.97	92.01	0.00

Supplementary Figure 11. 3D Mapping of A02 Binding Across the Binding Sites in the M^{pro} (Alternate Location B). AlphaSpace2 Pocket ID's, the corresponding M^{pro} binding sites, BScores, Alpha Space, and Occupancy are listed in companion table.

Supplementary Tables

*Supplementary Table 1***.** Summary of Original and Cleaned Datasets for AQUA, ESOL, and PHYS

Dataset	No. of Records in	
	Original	Cleaned
AQUA	1311	1311
ESOL	1128	1116
PHYS	2010	2001

Supplementary Table 2. Hyperparameter settings for the KANO models used for logS, logP, and M^{pro}-inhibitor prediction

**Hyperparameters**	**Values**
Initial learning rate	0.0001
Final learning rate	0.001
Numbers of layers	2
Batch size	256
Epoch	100
Ensemble size	5 (logS and logP), 10 (M^{pro}-inhibitor)
Activation function	ReLU
Metric	Accuracy (classification), RMSE (regression)
Threshold (classification)	0.5

Supplementary Table 3. KANO-M^{pro}-inhibitor Model Test Performance

	Precision	Recall	F1-score
Initial ratio	0.883	0.308	0.457
Resample 20%*	0.697	0.359	0.474
Resample 30%*	0.750	0.331	0.459
Resample 40%*	0.711	0.351	0.470
Resample 50%*	0.717	0.336	0.457
Resample 60%*	0.736	0.312	0.438
Resample 70%*	0.770	0.334	0.466
Resample 80%*	0.766	0.339	0.470
Resample 90%*	0.753	0.295	0.424
Resample 100%*	0.782	0.310	0.444

*Resample: First, undersample the inactive class by the specified percentage. Then, oversample the active class to achieve a 1:1 ratio of active to inactive samples.

Supplementary Table 4. Test Performance on OpenChem logP dataset.

Model	MAE	RMSE
KANO-logP	0.232 ± 0.003	0.366 ± 0.014
sPhysNet-MT-ens5[a]	0.242 ± 0.004	0.393 ± 0.012
QSPR[a]	-	0.78
GraphCNN[a]	-	0.56
DNN_{taut}[a]	-	0.47 ± 0.002
DNN_{mono}[a]	-	0.50 ± 0.002
OPERA[a]	-	0.78

[a]Values reported in Song's work.[1]

Supplementary Table 5. The Predicted Properties of the 24 Suggested Drug Candidates

Asinex id	Inhibition Probability	Predicted logP	Predicted logS	Tc
LAS 32402099	0.754	2.676	-4.652	0.31
BAS 15024440	0.682	3.038	-3.241	0.44
LEG 21432177	0.680	2.821	-4.029	0.29
ASN 13548051	0.678	3.165	-4.542	0.62
BDF 26216752	0.657	2.896	-4.209	0.36
BAS 12743765	0.654	3.225	-4.929	0.48
BDD 26205790	0.643	4.716	-4.597	0.35
BAS 01051463	0.635	2.210	-4.715	0.34
ADM 21433845	0.631	3.496	-4.878	0.36
ASN 05587292	0.631	3.013	-4.323	0.55
BDF 26220051	0.623	3.839	-3.925	0.34
IPE 22240583	0.621	2.004	-4.097	0.31
LAS 32402061	0.615	2.384	-4.750	0.31
BAS 12967408	0.612	3.459	-4.933	0.35
BDE 30875419	0.611	3.214	-4.260	0.39
BAS 00462047	0.609	3.142	-4.466	0.68
LEG 17130244	0.606	3.671	-4.244	0.34
BDC 27863502	0.581	4.248	-4.858	0.29
BDD 30726703	0.580	2.662	-3.832	0.39
BAS 04085488	0.580	2.572	-4.738	0.35
ADM 21434104	0.580	2.505	-3.761	0.3
ASN 05586220	0.578	3.880	-4.590	0.4
BAS 01860892	0.576	3.561	-4.760	0.35
BAS 02615914	0.574	2.775	-4.060	0.42

Supplementary Table 6. Structure determination and refinement parameters for A02-M^{pro} structure 9E9P.

	Crystal structure of SARS-CoV-2 Main Protease (Mpro) in Complex with Covalent Inhibitor A02 (PDB 9E9P)
**Data collection**	
Space group	C 1 2 1
Cell dimensions	
a, *b*, *c* (Å)	98.12, 81.11, 51.61
α, β, γ (°)	90.00, 114.80, 90.00
Resolution (Å)	29.98 – 1.76 (1.82 – 1.76)[a]
R_{merge}	0.091 (1.23)
$I / \sigma I$	8.20 (1.02)
$CC_{1/2}$	0.996 (0.517)
Completeness (%)	99.8 (99.5)
Multiplicity	4.4 (4.4)
**Refinement**	
Resolution (Å)	29.98 – 1.76
No. reflections	36360
R / R_{free}	0.174 / 0.211
No. non-H atoms	
Protein	2340
Ligand	36
Water	155
B-factors	
Protein	41.16
Ligand	55.34
Water	48.29
R.m.s. deviations	
Bond lengths (Å)	0.006
Bond angles (°)	0.92
Ramachandran plot statistics	
No. favored	297 (98.7 %)
No. allowed	4 (1.3 %)
No. outliers	0 (0.0 %)

Data set was collected from a single crystal. [a]Values in parentheses are for highest-resolution shell.

M^{pro}-inhibitor Dataset Collection:

The M^{pro}-inhibitor dataset is compiled from three primary sources. The first is the NIH 3CL enzymatic activity assay, which measures a compound's ability to inhibit 3CL protease, with 12,247 compounds in SMILES format. Inhibition levels range from 0% (with DMSO) to -100% (without enzyme). The second source is data collected in COVID Moonshot project, a global open-science initiative launched in March 2020, which collected 20,997 compounds (SMILES format), with 2,473 tested for inhibition. The standard measure for this project is IC_{50} in μM. The third data source involves the collection of experimentally tested compounds from over 40 research papers. To expand this dataset further, an extensive manual search is conducted across various journals. In total, 8,436 compounds with reported IC_{50} value have been collected. This comprehensive dataset provides a valuable resource for the study and development of M^{pro} drug discovery.

The data cleaning involves two steps: i. Unreadable SMILES Removal: To remove invalid (unreadable) SMILES strings, all SMILES are converted to standardized canonical SMILES using RDKit.[2] Any strings that fail to convert are removed. ii. Duplicated SMILES Checking: Additionally, to address duplicated SMILES entries with associated standard values (inhibition% or IC_{50}), the values are averaged. This data refinement ensures that the dataset is free of redundancy and that each unique SMILES corresponds to a single, standardized value.

In the M^{pro}-inhibitor dataset, classifying a compound's inhibition activity is key. Two thresholds were used for classification: for inhibition level, a -50% threshold resulted in 9,688 inactive and 163 active compounds in the NIH 3CL enzymatic activity assay. For IC50, a 10 µM threshold classified 8,055 compounds as inactive and 1,563 as active after data cleaning.

To check duplicated compounds and the correlation between different datasets, extra data filtration has been applied to the dataset. Compounds with inconsistent labels were removed to keep the consistency of the M^{pro} dataset.

Aqueous Solubility (logS) Dataset:

The three benchmark logS datasets first undergo a data cleaning process, which includes SMILES string standardization (details available at MolVS documentation). The standardization procedure generates unique, consistent SMILES representation for each molecule. Any records that fail to pass the standardization are removed. The number of data records before and after data cleaning is presented in Supplementary Table 1.

KANO-M^{pro}-Inhibitor Model

The M^{pro}-inhibitor dataset is highly imbalanced, with 12.2% active and 87.8% inactive compounds. To address this imbalance, we explored different training strategies. These included training with the initial dataset distribution, which reflects the real-world scenario but may lead to a biased model; undersampling the inactive class at various ratios to balance the class distribution, which could improve the model's ability to identify active compounds but risks losing valuable information from the majority class; and oversampling the active class data to increase its representation, which helps prevent the model from overlooking active compounds but may introduce overfitting. Each strategy was carefully evaluated to determine its impact on model performance. The result is shown in Supplementary Table 3.

KANO-logS Model

For duplicate and overlapping entries between AQUA, ESOL, and PHYS datasets, we averaged logS values for model training. We first assess whether combining datasets improves model generalizability by using overlapping records as the training/validation set (training:validation = 4:1 ratio) and unique records as the test set (Supplementary Figure 5). Additionally, we train the model with a random 8:1:1 split, with results shown in Supplementary Figure 7.

KANO-logP model

We randomly split the OpenChem logP dataset into training, validation, and testing sets of 11,340, 1,418, and 1,418 samples, respectively, using 5 different random seeds. For each seed, 5 models were trained, and the average performance of the ensemble predictions is used to further improve the model performance.

Reference

(1) Xia, S.; Zhang, D.; Zhang, Y. Multitask Deep Ensemble Prediction of Molecular Energetics in Solution: From Quantum Mechanics to Experimental Properties. *Journal of Chemical Theory and Computation* **2023**, *19* (2), 659-668. DOI: 10.1021/acs.jctc.2c01024.
(2) RDKit: Open-source cheminformatics. https://www.rdkit.org (accessed 2022）.
